# Cortical Midline Structures and Autobiographical-Self Processes: An Activation-Likelihood Estimation Meta-Analysis

**DOI:** 10.3389/fnhum.2013.00548

**Published:** 2013-09-04

**Authors:** Helder F. Araujo, Jonas Kaplan, Antonio Damasio

**Affiliations:** ^1^Brain and Creativity Institute, University of Southern California, Los Angeles, CA, USA; ^2^Neuroscience Graduate Program, University of Southern California, Los Angeles, CA, USA; ^3^Graduate Program in Areas of Basic and Applied Biology, University of Oporto, Oporto, Portugal

**Keywords:** autobiographical-self, autobiographical memory, cortical midline structures, meta-analysis, fMRI, self

## Abstract

The autobiographical-self refers to a mental state derived from the retrieval and assembly of memories regarding one’s biography. The process of retrieval and assembly, which can focus on biographical facts or personality traits or some combination thereof, is likely to vary according to the domain chosen for an experiment. To date, the investigation of the neural basis of this process has largely focused on the domain of personality traits using paradigms that contrasted the evaluation of one’s traits (self-traits) with those of another person’s (other-traits). This has led to the suggestion that cortical midline structures (CMSs) are specifically related to self states. Here, with the goal of testing this suggestion, we conducted activation-likelihood estimation (ALE) meta-analyses based on data from 28 neuroimaging studies. The ALE results show that both self-traits and other-traits engage CMSs; however, the engagement of medial prefrontal cortex is greater for self-traits than for other-traits, while the posteromedial cortex is more engaged for other-traits than for self-traits. These findings suggest that the involvement CMSs is not specific to the evaluation of one’s own traits, but also occurs during the evaluation of another person’s traits.

## Introduction

The autobiographical-self can be described as a mental state deriving from a momentary access to information regarding facts and events in one’s life (Damasio, 1998). The access depends on the retrieval and assembly of memories pertaining to a multitude of facts and events and is likely to vary with the kinds of memories involved. Access may focus on retrieval of relatively simple memory representations, as when one retrieves information regarding demographic aspects of one’s identity (e.g., one’s nationality); or it may be more specific and involve retrieval of representations of perceptual and emotional aspects of a particular episode (e.g., one’s college graduation). The effort needed for the retrieval is likely to vary as well and it is probably smaller for memories pertaining to prominent aspects of one’s biography than for memories regarding more remote events. Once memories are displayed, they may trigger a varied amount of related memories and the associated emotional responses. In brief, the nature and scope of the knowledge exhibited in an autobiographical-self state varies according to the domains of information that are recruited.

The investigation of the behavioral and neural correlates of the autobiographical-self has explored varied domains, including one’s own name (e.g., Tacikowski et al., 2011), voice (e.g., Nakamura et al., 2001), body parts (e.g., Platek et al., 2008) and personality traits (e.g., Kelley et al., 2002), and autobiographical memories (e.g., Cabeza and St Jacques, 2007). Here, we focus on the domain of personality traits. By contrasting self-traits (i.e., deciding if a given personality trait accurately describes oneself) with other-traits (i.e., deciding if a given personality trait accurately describes another person), some studies have found an advantage of self-traits over other-traits in terms of reaction times (RTs) and memory performance. This has led to the suggestion that information pertaining to self is processed differently from information pertaining to another person, and has become known as the “self-referent effect” (Rogers et al., 1977). Moreover, it has led to the idea that the neural basis of self-reference involves cortical midline structures (CMSs), namely the medial prefrontal cortex (MPFC), anterior cingulate cortex (ACC), and posteromedial cortices (PMCs) (reviewed in Northoff et al., 2006). The results of the existing studies are not conclusive, however, in regard to the existence of the self-referent effect (e.g., Symons and Johnson, 1997) as well as in regard to the association of CMSs with self-reference (e.g., Legrand and Ruby, 2009).

With the development of techniques capable of performing meta-analysis of neuroimaging data, some attempts have been made to investigate consistent differences between self and other in terms of brain activity (Northoff et al., 2006; Qin and Northoff, 2011; Qin et al., 2011; Denny et al., 2012). Although informative, the studies included in these meta-analyses varied in terms of the self-referential stimuli used (comprising, for example, autobiographical and episodic memories, personality traits, the participants’ faces or other body parts, and the participants’ names), as well as in terms of the tasks performed (including, for example, tasks in which the participants were not given any specific instructions other than to look at or to listen to the stimuli; and tasks in which the participants were asked to judge/evaluate or to reflect on aspects of the stimuli). This heterogeneity of domains and approaches is a potential limitation given that autobiographical-self processes are likely to vary according to the stimuli and the tasks one uses (as discussed in Klein and Gangi, 2010). In addition, the kinds of “other” used in the original study and the relationship between self and other are likely to be decisive in establishing differences between self and other. The differences between self and other in terms of RTs and memory performance have been shown to be reduced or eliminated when the other is a close acquaintance, such as the participants’ close friends (Symons and Johnson, 1997), or parents (Markus and Kitayama, 1991). In addition, activation in CMSs seems to vary according to who the other is. For example, activity in the MPFC during evaluation of traits for self is not different from that of a close other, but happens to be greater for self than for a distant other (Ochsner et al., 2005).

Here, we conduct meta-analyses of the previously reported brain activations restricted to the direct evaluation of personality traits pertaining to self (“self-traits”) and to other (“other-traits”). We attempt to compare self and other in regard to processes underlying equivalent tasks with equivalent stimuli. We also investigate how the contrast of brain activity between self-traits and other-traits varied according to who the other is in relation to self (distant others versus close others).

Our working assumption is that in order to evaluate when a given personality trait describes one’s self accurately, one needs to retrieve and assemble memories (an autobiographical-self state) and decide based on the knowledge accessed. These processes are likely to depend on structures capable of high-levels of integration, such as CMSs (Parvizi et al., 2006; Hagmann et al., 2008); they may also engage structures involved in emotion-related somatic representations such as the insula because of the subjective and emotional content of the personality traits (Damasio and Carvalho, 2013). Furthermore, evaluating when a given personality trait describes another person requires memory retrieval and decisions and is thus likely to involve similar brain structures. Nonetheless, we predict differences between self and other in terms of brain activity. These differences are probably commensurate with the differences present in the representations accessed during the evaluation. Representations regarding one’s self are elaborated during a lifetime of episodes and events, whereas representations regarding another person are probably elaborated via a more limited amount of interactions with that person during the acquaintanceship. Thus the representations regarding one’s self are probably more numerous and more easily retrieved than those regarding another person, and it is also probable that emotion responses associated with the evaluation are greater for self than for other. Finally, the differences between self and other may be greater when the other is a distant other than when the other is a close other, someone with whom one has a close relationship and interacts frequently over a long period of time.

## Methods

### Studies used

The studies included were found and retrieved via PubMed and PsychARTICLES, using “self” as a search word for studies that used functional magnetic imaging (fMRI). The citations within the retrieved publications were also explored as possible studies to include in the meta-analysis. This initial search was concluded by November 31, 2012. From the initial pool of retrieved publications, we selected only studies that investigated the direct evaluation of the domain of personality traits regarding self (i.e., the participants were asked to judge whether a set of personality traits described themselves), other (i.e., the participants were asked to judge whether a set of personality traits described another person), or both. We restricted the selection to studies that presented whole-brain analyses and included healthy subjects whose ages ranged from 18 to 50 years old.

The final selection assembled 28 publications, 31 studies (each study including a different set of participants; Table 1). We categorized the kind of other used in the experiments into two groups: (i) *distant others*, which included a well-known person from the public domain (e.g., former US President George W Bush); or a distant acquaintance of the subject (e.g., a classmate); (ii) *close others*, which included friends, siblings or romantic partners, or the participants’ parents. Data regarding other-traits for underrepresented categories of other (i.e., Harry Potter in Pfeifer et al., 2007, and historic religious leaders in Han et al., 2010) were not included in the analysis.

**Table 1:** **Individual experiments included in the meta-analysis**.

Study	Number of subjects	Self > baseline	Other > baseline	Self > other	Other > self
Benoit et al. (2010)	16	1	1	1	1
D’Argembeau et al. (2007)	17	0	0	1	0
D’Argembeau et al. (2010)	20	1	1	0	0
Fossati et al. (2003)	14	1	0	0	0
Gutchess et al. (2007)	19	0	0	1	1
Han et al. (2010)	14	1	0	1	0
Heatherton (2006)	30	1	1	1	1
Jenkins and Mitchell (2011)	15	0	0	1	1
Kelley et al. (2002)	20	0	0	1	0
McAdams and Krawczyk (2012)	18	0	0	1	1
Modinos et al. (2009)	16	0	0	1	1
Modinos et al. (2011)	18	1	1	1	1
Murphy et al. (2010)	10	1	1	0	1
Ochsner et al. (2005)	17	1	1	0	0
Ochsner et al. (2005)	16	0	0	2	2
Pfeifer et al. (2009)	17	1	0	0	0
Pfeifer et al. (2007)	17	1	0	0	0
Powell et al. (2009)	28	0	0	1	1
Schmitz et al. (2004)	19	1	1	1	0
Schmitz and Johnson (2006)	15	1	0	0	0
van Buuren et al. (2010)	19	1	0	0	0
Vanderwal et al. (2008)	17	0	1	1	1
Wang et al. (2012)	32	1	3	3	0
Whitfield-Gabrieli et al. (2011)	10	1	0	0	0
Yaoi et al. (2009)	17	1	1	0	0
Yoshimura et al. (2009)	15	1	1	1	1
Zhang et al. (2006)	7	1	0	1	0
Zhang et al. (2006)	7	1	0	1	0
Zhu et al. (2007)	13	1	2	1	0
Zhu et al. (2007)	13	1	2	2	0
Zhu et al. (2012)	14	0	0	1	0

*The same study is listed twice when it included two different populations*.

The coordinates of the peaks of activation foci were recorded for each contrast in each experiment. Foci referring to the same contrast of interest (e.g., other > baseline) that derived from more than one experiment (e.g., distant other and the participant’s mother) using the same group of participants, were analyzed together (for that contrast) in order to minimize within-group effects (Turkeltaub et al., 2011). The total number of foci, experiments, and participants for each contrast were as follows: (i) *self-traits* > *baseline*, 159 foci, 21 experiments, 340 participants; (ii) *other-traits* > *baseline*, 114 foci, 12 experiments, 219 participants for both distant and close others; 46 foci, 6 experiments, 95 participants for distant others; and 68 foci, 9 experiments, and 167 participants; (iii) *self-traits* > *other-traits*, 148 foci, 22 experiments, 383 participants for both distant others and close others; 98 foci, 15 experiments, 259 participants for distant others; 50 foci, 10 experiments, 185 participants for close others; (iv) *other-traits* > *self-traits*, 61 foci, 12 experiments, 218 participants, for distant others and close others combined; 23 foci, 7 experiments, 127 participants, for distant others; 38 foci, 6 experiments, 107 participants, for close others.

The baseline included in the studies was either rest (three experiments regarding self-traits > baseline) or an active task involving some judgment of trait words, such as in relation to the number of syllables of the words, the case, or the font in which the words were written, the valence of the words (17 experiments regarding self-traits > baseline; and all the experiments regarding other-traits > baseline).

Data regarding the RTs were also recorded; these data were available in 15 experiments: 9 referring to experiments that involved distant others, and 6 referring to experiments that involved close others.

### Data analysis

A probabilistic map of activation was generated for each contrast of interest using activation-likelihood estimate (ALE) with GingerALE2.3^1^. The steps involved in this estimation are explained in detail in Turkeltaub et al. (2011). For a given contrast, the ALE values represent the likelihood of observing activity in that voxel for at least one group of participants (Turkeltaub et al., 2011). The coordinates in Talairach were transformed into MNI (SPM) using icbm2tal transform (Lancaster et al., 2007; Laird et al., 2010). Two thresholds were applied to the results: first, a threshold of *p* < 0.001 uncorrected; subsequently, a cluster size probability threshold of *p* < 0.05 determined by permutations of random data (5000 permutations). The ALE maps were compared between contrasts of interest using the ALE subtraction analysis (random effects, Laird et al., 2005) available in the same software. This included a permutation test (5,000 permutations) to determine the statistical significance of the differences, and a threshold of *p* < 0.001 (uncorrected). All the results are in MNI coordinates and were overlaid in a standard MNI brain (Colin27_T1_seg_MNI.nii) using Mango^2^ and MRIcroGL^3^.

The effect size for the difference in RT between self and other was assessed using the reported *t-*test and *F-*test parameters, and calculating point-biserial correlation *r* values, as suggested and explained in Rosenthal and DiMatteo (2001). In brief, the *r* values were calculated using the following formula: *r* = [*t*^2^/(*t*^2^ + df)]^1/2^, or *r* = [*F*^2^/(*F*^2^ + df_error_)]^1/2^. Then, the *r* values were converted into *Fisher Z* values; mean *Z* scores and corresponding 95% confidence interval were calculated for the experiments according to the kind of other (close others and distant others), and then transformed back into *r* values.

## Results

### Reaction times

Reaction times tended to be greater for other-traits than for self-traits. The average unstandardized difference between mean RTs for other-traits and mean RTs for self-traits was 24.53 ms (SEM = 12.56 ms; mean RTs reported in 13 experiments). Statistically significant differences between self-traits and other-traits were reported in six experiments (five regarding distant others, and 1 regarding close others); in five of these experiments (four referring to distant others and 1 referring to close others), mean RTs were greater for other than for self.

The average unstandardized difference between mean RT for other-traits and mean RT for self-traits was greater when addressing distant others (*M* = 32.93 ms; SEM = 17.98 ms; *N* = 8 experiments) than when addressing close others (*M* = 11.10 ms; SEM = 15.9 ms; *N* = 5 experiments). The 95% confidence interval of the effect size *r* followed the same trend: for distant others, it was 0.897 ± 0.804 ms (*N* = 7 experiments); for close others, it was 0.299 ± 0.202 ms (*N* = 6 experiments).

### Meta-analyses of brain activation

#### Self-traits versus baseline

**Figure 1:**
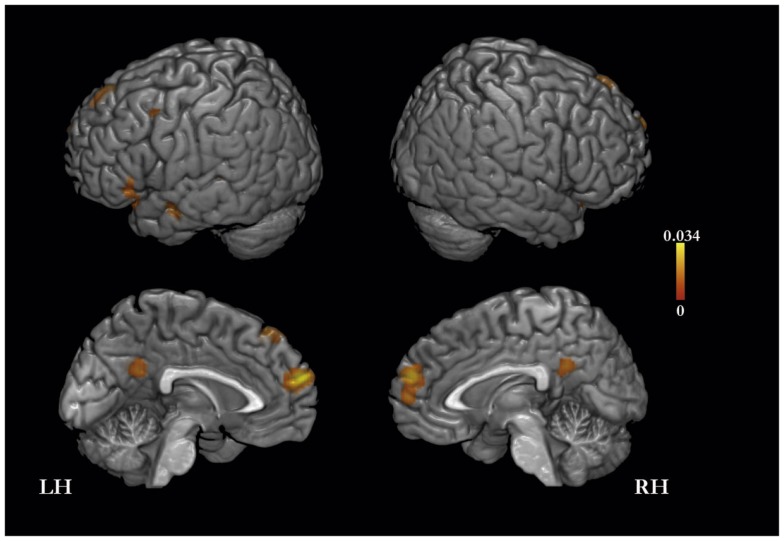
**Meta-analysis of activation foci (159 foci; 21 experiments) for self-traits compared with baseline**.

The meta-analysis of activation foci for self-traits yielded eight clusters of significant activation-likelihood (ALE): bilaterally in MPFC, PMC, and lateral prefrontal cortex, and in the left insula and middle temporal gyrus (Table 2; Figure 1).

**Table 2:** **Meta-analysis of activation foci for self-traits compared with baseline (159 foci; 21 experiments)**.

Cluster no.	Brain region	*x*	*y*	*z*	Volume (mm^3^)	ALE (×10^−3^)
1	L medial prefrontal cortex	−2	60	22	5152	33.97
2	L insula/inferior frontal gyrus	−34	20	−12	3168	21.20
3	L posteromedial cortex	−4	−52	26	2304	17.77
4	L superior frontal gyrus	−8	36	50	1744	19.56
5	L middle temporal gyrus	−60	−4	−16	880	14.71
6	L supramarginal gyrus	−44	−54	28	808	21.09
7	R inferior frontal gyrus	48	26	−14	488	14.17
8	L middle temporal gyrus	−60	−36	2	440	14.65
9	L middle frontal gyrus	−42	8	48	440	14.96

#### Other-traits versus baseline

**Figure 2:**
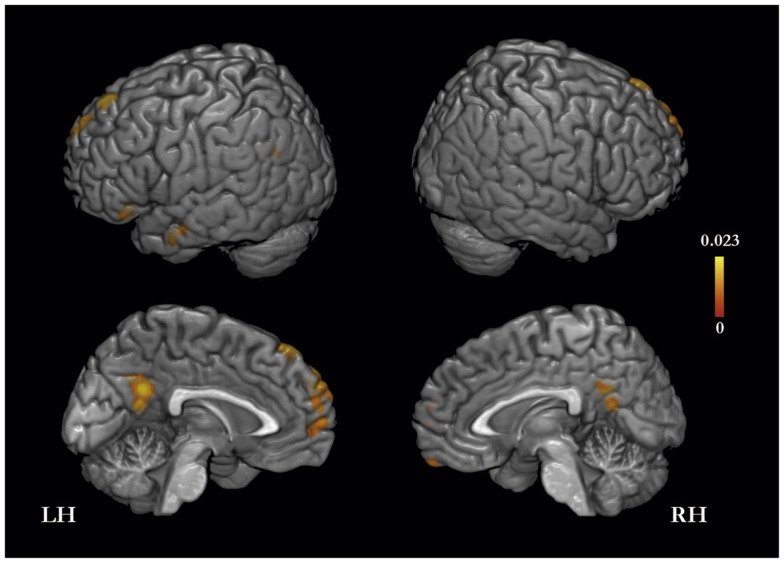
**Meta-analysis of activation foci (114 foci; 12 experiments) for other-traits compared with baseline**.

The meta-analysis of activation foci for other-traits regarding distant and close kinds of other yielded eight clusters of significant ALE: bilaterally, in the MPFC and PMC, in the left inferior frontal, middle temporal, and angular gyri, and in the right orbitofrontal gyrus (Table 3; Figure 2). The same meta-analysis restricted to distant others (i.e., a category that includes a well-know person of the public domain or participants’ distant acquaintances such as classmates or housemates) revealed 24 clusters of significant ALE: bilaterally in the PMC, MPFC, middle temporal and supramarginal gyri, and in the left superior frontal gyrus and temporal pole, and in the right orbitofrontal gyrus and cerebellum (Table 3). In addition, the same meta-analysis restricted to close others (i.e., a category that includes a close acquaintance or relative of the participants, such as the participants’ parents, or a participant’s best friend/or sibling) yielded six clusters of significant ALE: bilaterally in the MPFC and PMC, and in the left superior and inferior frontal gyri and middle temporal gyrus (Table 3).

**Table 3:** **Meta-analysis of activation foci for other-traits compared with baseline in relation to both kinds of other (114 foci; 12 experiments), to distant others (46 foci; 6 experiments), and to close others (68 foci; 9 experiments)**.

Cluster no.	Brain region	*x*	*y*	*z*	Volume (mm^3^)	ALE (×10^−3^)
**OTHER-TRAITS > BASELINE**						
**Distant and close others**
1	L superior frontal gyrus/medial prefrontal cortex	−10	56	32	3544	16.10
2	L posteromedial cortex	−4	−54	28	3160	23.14
3	L superior frontal gyrus	−10	42	48	1632	20.20
4	L inferior frontal gyrus	−48	28	−8	976	15.67
5	L middle temporal gyrus	−60	−12	−14	968	13.18
6	L angular gyrus	−50	−66	28	616	10.60
7	L temporal pole	−44	10	−36	528	12.09
8	R orbitofrontal gyrus	6	58	−24	336	12.96
**Distant others**
1	L middle temporal gyrus	−60	0	−26	1224	12.73
2	L medial prefrontal/superior frontal gyrus	−6	60	28	696	10.90
3	L posteromedial cortex	−4	−56	30	632	10.18
4	R medial prefrontal cortex	2	46	−20	552	9.75
5	L temporal pole	−42	10	−38	448	10.68
6	L supramarginal gyrus	−48	−64	34	288	8.94
7	R temporal pole	48	12	−28	96	8.61
8	L temporal pole	−54	2	−38	64	8.05
9	L medial prefrontal cortex	−8	52	−2	56	8.17
10	L frontal pole/medial prefrontal cortex	−2	64	4	56	8.17
11	R/L posteromedial cortex	0	−56	16	56	8.32
12	R cerebellum	32	−84	−32	48	7.77
13	R temporal pole/middle temporal gyrus	58	10	−26	48	7.49
14	R middle temporal gyrus	66	−4	−20	48	7.63
15	L superior frontal gyrus	−12	34	50	48	7.81
16	L superior frontal gyrus	−14	46	50	48	7.74
17	L temporal pole	−44	22	−42	40	7.44
18	L medial prefrontal cortex	−8	36	−20	40	7.82
19	R posteromedial cortex	8	−42	30	40	7.73
20	L superior frontal gyrus	−10	54	42	40	8.00
21	R orbitofrontal cortex	6	58	−26	32	7.45
22	R middle temporal gyrus	58	−12	−20	32	7.38
23	R supramarginal gyrus	58	−58	16	32	7.52
24	R posteromedial cortex	12	−46	26	32	7.41
**Close others**
1	L superior frontal gyrus	−10	42	48	1424	20.20
2	L posteromedial cortex	−4	−54	28	1328	14.80
3	L inferior frontal gyrus	−48	28	−8	1136	15.67
4	L medial prefrontal cortex/superior frontal gyrus	−12	56	32	624	13.42
5	L middle temporal gyrus	−62	−30	−2	552	11.31
6	L medial prefrontal cortex/superior frontal gyrus	−2	58	18	352	10.16

### Self-traits versus other-traits

#### Self-traits versus other-traits for both distant others and close others

**Figure 3:**
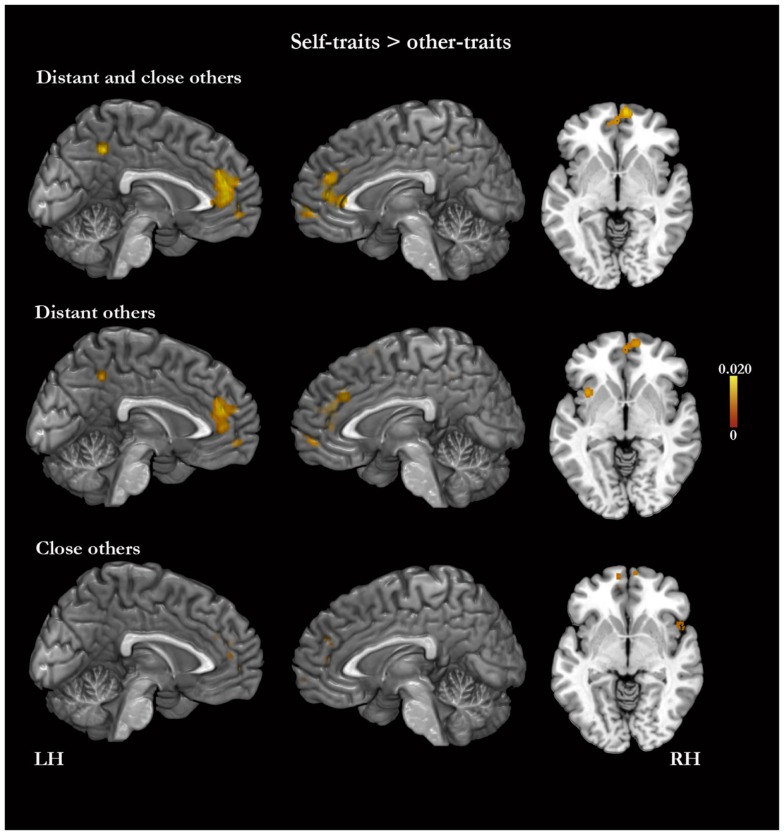
**Meta-analysis of activation foci for self-traits compared with other-traits in relation to both kinds of other (148 foci; 22 experiments), to distant kinds of other (98 foci; 15 experiments), and to close kinds of other (50 foci; 10 experiments)**.

**Figure 4:**
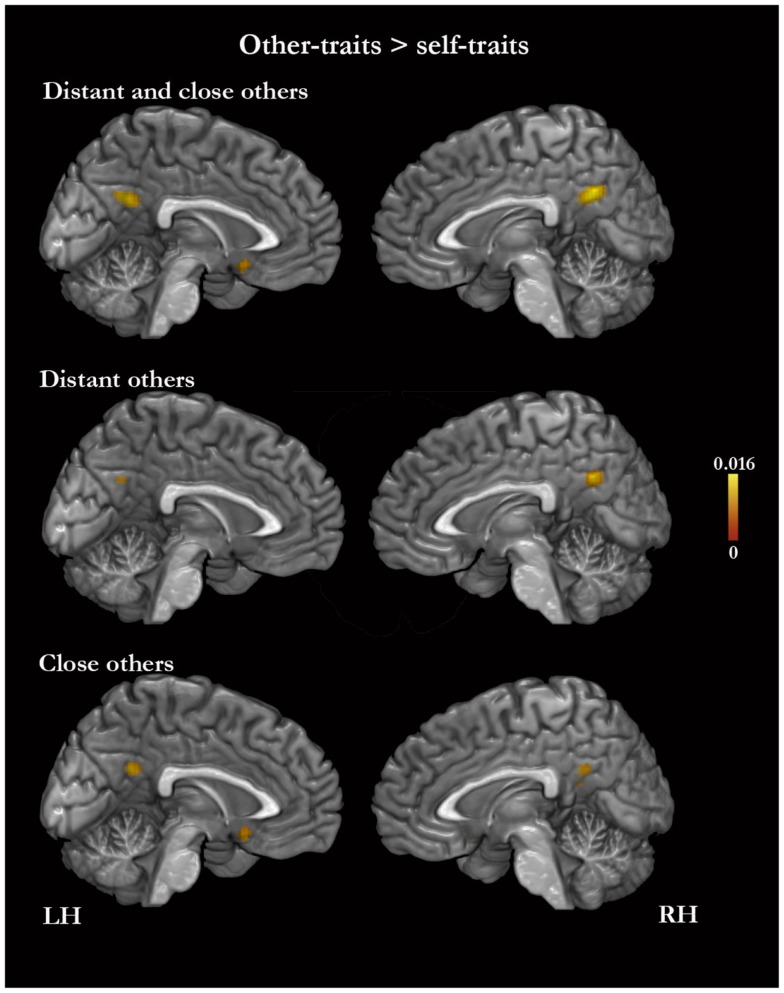
**Meta-analysis of activation foci for other-traits compared with self-traits in relation to both kinds of other combined (61 foci; 12 experiments), to distant kinds of other (23 foci; 7 experiments), and to close kinds of other (38 foci; 6 experiments)**.

In the meta-analysis of the activation foci for self-traits > other-traits, we observed four clusters of significant ALE: bilaterally, in the MPFC and ACC, in the left PMC, and in the right middle frontal gyrus (Table 4; Figure 3). The meta-analysis of the activations relative to the reverse contrast (other-traits > self-traits) yielded eight clusters of significant ALE: bilaterally in the PMC and medial temporal gyrus, and in the right basal forebrain, superior parietal lobule, and cerebellum (Table 5; Figure 4).

**Table 4:** **Meta-analysis of activation foci for self-traits compared with other-traits in relation to both kinds of other (148 foci; 22 experiments), to distant kinds of other (98 foci; 15 experiments), and to close kinds of other (50 foci; 10 experiments)**.

Cluster no.	Brain region	*x*	*y*	*z*	Volume (mm^3^)	ALE (×10^−3^)
**SELF-TRAITS > OTHER-TRAITS**						
**Distant and close others**
1	L medial prefrontal cortex/anterior cingulate cortex	−6	46	20	8296	20.06
2	L superior frontal gyrus/middle frontal gyrus	−22	52	30	1488	18.17
3	R middle frontal gyrus	28	52	26	736	14.52
4	L posteromedial cortex	−4	−50	46	584	18.47
**Distant others**
1	R medial prefrontal cortex	8	32	30	3696	16.61
2	R superior frontal gyrus	−22	52	30	1384	18.16
3	R medial prefrontal cortex	10	58	−6	648	12.24
4	R superior frontal gyrus	−22	40	40	456	11.42
5	R superior frontal gyrus/premotor cortex	10	12	64	416	13.00
6	L insula	−38	20	4	384	14.22
7	L angular gyrus	−56	−48	20	368	13.23
8	L posteromedial cortex	−4	−48	46	360	12.51
9	L insula	−36	12	−6	336	12.80
**Close others**
1	R medial prefrontal cortex	8	42	24	520	14.31
2	L medial prefrontal cortex	−8	46	20	480	13.67
3	L medial prefrontal cortex	−8	34	24	448	12.67
4	L medial prefrontal cortex	−8	50	−2	448	12.36
5	R insula/inferior frontal gyrus	50	16	−10	328	10.42
6	R anterior cingulate cortex medial prefrontal cortex	14	42	4	328	10.32
7	L superior frontal gyrus	−8	60	−6	96	8.92
8	R medial prefrontal cortex	2	44	10	96	8.87
9	L occipital lateral gyrus	−50	−72	−12	80	8.92
10	R middle frontal gyrus	26	52	16	80	8.60
11	R superior frontal gyrus/frontal pole	8	64	−8	72	8.91
12	R superior frontal gyrus	14	32	52	72	8.89
13	R middle frontal gyrus	64	−38	−2	64	8.43

**Table 5:** **Meta-analysis of activation foci for other-traits compared with self-traits in relation to both kinds of other combined (61 foci; 12 experiments), to distant kinds of other (23 foci; 7 experiments), and to close kinds of other (38 foci; 6 experiments)**.

Cluster no.	Brain region	*x*	*y*	*z*	Volume (mm^3^)	ALE (×10^−3^)
**OTHER-TRAITS > SELF-TRAITS**						
**Distant and close others**
1	R posteromedial cortex	4	−58	30	1208	16.26
2	L medial temporal gyrus	−58	−16	−22	672	12.90
3	R medial temporal gyrus	48	−16	−22	296	11.19
4	R basal forebrain	−2	14	−14	288	9.88
5	R superior parietal lobule	22	−66	54	120	8.29
6	R cerebellum	18	−52	−28	80	8.89
7	R superior parietal lobule	−40	−56	52	64	8.64
8	R middle temporal gyrus	−48	30	−14	56	9.06
**Distant others**
1	R posteromedial prefrontal	4	−60	30	384	13.3
2	L medial temporal gyrus	−62	−8	−26	56	9.26
**Close others**
1	L/R posteromedial cortex	0	−52	26	456	11.73
2	L basal forebrain	−2	14	−14	352	9.88
3	R cerebellum	18	−52	−28	96	8.89
4	L superior parietal lobule	−40	−56	52	96	8.64
5	L posteromedial cortex	6	−50	18	56	8.30
6	R posteromedial cortex	−12	−58	22	56	8.17

#### Self-traits versus other-traits for distant others

The meta-analysis of the activation foci for self-traits > other-traits regarding only distant others yielded nine clusters of significant ALE, namely, bilaterally, in the MPFC, in the right superior frontal gyrus, and in the left PMC, insula, and angular gyrus (Table 4; Figure 3). The meta-analysis of activation foci regarding the reverse contrast (other-traits > self-traits) rendered two clusters of significant ALE in, bilaterally, the PMC and in the left middle temporal gyrus (Table 5; Figure 4).

#### Self-traits versus other-traits for close others

The meta-analysis of the activation foci for self-traits > other-traits for only close others revealed clusters of volumes greater than 100 mm^3^ bilaterally in the MPFC. In addition, one of the clusters we identified falls outside of the standard brain, but in proximity to the left insula/inferior frontal gyrus. Also, the same analysis yielded additional clusters of significant ALE with smaller volumes, namely, in the lateral prefrontal, temporal, and occipital lobes (Table 4; Figure 3). The meta-analysis of activations for the reverse contrast (other-traits > self-traits) revealed two clusters of volumes greater than 100 mm^3^, bilaterally, in the PMC and in the right basal forebrain, and clusters with smaller volumes, bilaterally, in the PMC, in the right cerebellum, and in the left superior parietal lobule (Table 5; Figure 4).

### Comparisons between contrasts (subtraction analyses)

#### Other-traits > baseline for close others versus other-traits > baseline for distant others

A subtraction analysis did not yield differences of ALE results for other-traits > baseline between close others and distant others. A conjunction analysis revealed an overlap of ALE scores for other-traits > baseline between the two kinds of other in a large cluster in the PMC (cluster 1 – MNI coordinates: −3, −54, −29; ALE: 10.2; volume: 384 mm^3^) as well as in smaller clusters in the left superior frontal gyrus (cluster 2 – MNI coordinates: −13, 45, 51; ALE: 7, 54; volume: 40 mm^3^; cluster 3 – MNI coordinates: −11, 35, 50; ALE: 7, 51; volume: 32 mm^3^) and in bilaterally in the PMC (cluster 4: MNI coordinates: −8, −57, −30; ALE: 7, 74; volume: 16 mm^3^; cluster 5: MNI coordinates: 0, −56, 18; ALE: 7, 43; volume: 8 mm^3^).

#### Self-traits > other-traits for close others versus self-traits > other-traits for distant others

In a subtraction analysis, ALE results for self-traits > other-traits regarding close others were not different from those regarding distant others. Nonetheless, a conjunction analysis revealed an overlap of ALE results for self-traits > other-traits between the two kinds of others in three clusters in the MPFC/ACC (cluster 1 – MNI coordinates: −5, 45, 20; ALE: 9.8; volume: 112 mm^3^; cluster 2 – MNI coordinates: 0, 44, 9; ALE: 8.7; volume: 40 mm^3^; cluster 3 – MNI coordinates: −5, 37, 24; ALE: 8.2; volume: 24 mm^3^) and one cluster in the frontal pole (cluster 4 – MNI coordinates: 8, 62, −6; ALE: 7.65; volume: 8 mm^3^).

#### Other-traits regarding close others > self-traits versus other-traits regarding distant others > self-traits

A subtraction analysis did not yield differences of ALE results for other-traits > self-traits between close others and distant others. In addition, a conjunction analysis showed an overlap of ALE results (for other-traits > self-traits) between the two kinds of other in a cluster in the PMC (MNI coordinates: 2, −56, 29; ALE; 7.1; volume = 16 mm^3^).

## Discussion

The processes of memory retrieval and decision that support the evaluation of one’s personality traits vary depending on the recalled material. For example, it has been shown that both behavioral measures and brain activity during the evaluation of one’s traits depend on how relevant the trait is to the individual’s identity (e.g., Markus, 1977; Kuiper, 1981; Lieberman et al., 2004). The same factors are also likely to play a role in the evaluation of traits pertaining to another person and possibly account, at least in part, for the varied results reviewed in the published studies. Still, a meta-analysis of those published data may help us gain a better perspective on the problem.

The results of the present meta-analyses reveal similarities and differences between self-traits and other-traits in terms of activation foci. Contrasted with baseline, self-traits and other-traits engage some of the same brain structures, including CMSs such as the MPFC and the PMC. Nonetheless, the results also reveal parametric differences between self and other in terms of activation in CMSs as well as in the insula and basal forebrain. The ALE results, referring to the contrast of other-traits with baseline and to the contrasts between other-traits and self-traits, seem to indicate that these differences may depend on the kind of other on which the study focused. We note, however, that the subtraction analyses did not confirm an effect of the type of other in any of the contrasts.

The MPFC and PMC are important hubs of brain connectivity and are presumably capable of high-levels of integration (Parvizi et al., 2006; Hagmann et al., 2008). They are known to exhibit greater activation during rest and during passive tasks than during a variety of demanding exteroceptive tasks (reviewed in Buckner et al., 2008). This suggests that these regions are preferentially involved in processing recalled, internally generated representations, something that is supported by their significant involvement during mind wandering (Mason et al., 2007), lapses of attention in externally oriented tasks (Weissman et al., 2006), and imagining future events (Schacter et al., 2012). We believe that their engagement in the evaluation of personality traits relates to retrieval and assembly of memories and to involvement in decision processes. Moreover, although the MPFC and PMC are interconnected and frequently activated during some of the same tasks, it is probable that these structures differ from each other in terms of the scope of representations they process.

The data derived from our meta-analyses show that the MPFC is generally more active for self-traits than for other-traits, and, although not confirmed by the subtraction analysis, this difference seems to be greater in the case of a distant other than a close other. There is strong evidence that MPFC is involved in the participation of somatic signals in processes of decision-making (Bechara et al., 2000a,b). It is thus possible that the differences of MPFC activity relate to emotion-related somatic representations in response to the memories retrieved and the decision. These responses are probably greater for self-traits than for other-traits but the difference is possibly smaller when referring to a close other than when referring to a distant other. We note that the differences between self-traits and other-traits in terms of insula activity are commensurate with those found for MPFC activity. In addition, it is also possible that the MPFC may be particularly involved in memory retrieval, namely by processing perceptual and somatic representations of the memories retrieved and thus contributing to a so-called “felt-rightness” during the retrieval (Moscovitch and Winocur, 2002). As discussed earlier, individuals are likely to have greater amount of memories for self than for another person; moreover, the memories are also likely to contain a greater amount of information, including both perceptual and somatic, when they pertain to self than when they pertain to another person. These differences are probably greater for a distant other than for a close other.

Intriguingly, our meta-analyses show that the PMC is more active for other-traits than for self-traits. The analyses relative to the contrast other-traits > self-traits derive from a smaller number of experiments than those regarding the opposite contrast, and this may limit the related statistical power. Nonetheless, we believe that the differences of PMC activity relate to effort in memory retrieval. The representations that regard self are probably more efficiently retrieved than those regarding another person, as supported by data regarding the RTs. Greater effort would translate into greater PMC activity. It is possible that by abstracting from episodes and facts during their lives, individuals have preassembled summary representations for some of their own personality traits (Klein and Loftus, 1993). It is also possible that individuals hold similar summary representations for aspects of their acquaintances’ personalities although that is more likely to occur in the case of close acquaintances than distant ones (Fuhrman and Funder, 1995).

There is indeed evidence for involvement of the PMC in memory retrieval both for information that regards self and for information that regards other people or things (e.g., Wagner et al., 2005; Binder et al., 2009; Rissman and Wagner, 2012). In addition, it has been shown that activity in the PMC relates to the retrieval effort. For example, the PMC shows greater activity during the recall of information than during the repetition of information (Buckner et al., 1996; Schacter et al., 1996).

It is likely that sub-areas within the same CMS are differently activated in different conditions. For example, although the PMC is generally more active for other-traits, it shows also a cluster of greater activity for self-traits than for other-traits in the present meta-analysis. It has also been proposed that the MPFC is differentially activated by self and other, with the most ventral areas more active for self and more dorsal areas more active for other (reviewed in Amodio and Frith, 2006).

In conclusion, our results provide evidence that self-traits and other-traits may depend on the same brain structures, including CMSs. Moreover, the differences between self-traits and other-traits vary according to who the other is in relation to self. We believe that these findings are linked to processes of memory retrieval and decision that underlie the evaluation of personality traits.

## Conflict of Interest Statement

The authors declare that the research was conducted in the absence of any commercial or financial relationships that could be construed as a potential conflict of interest.

## Footnotes

^1^http://brainmap.org/ale/index.html

^2^http://ric.uthscsa.edu/mango/

^3^http://www.mccauslandcenter.sc.edu/mricrogl/
